# Evidence from NHANES 2011–2014: a correlation between the weight-adjusted-waist index and cognitive abilities in the United States

**DOI:** 10.3389/fnagi.2025.1480609

**Published:** 2025-01-30

**Authors:** Chan Zhao, Xintian Xu, Chunyan Hao

**Affiliations:** ^1^First Hospital of Shanxi Medical University, Taiyuan, Shanxi, China; ^2^Yuncheng First Hospital, Yuncheng, China

**Keywords:** National Health and Nutrition Examination Survey (NHANES), cognitive ability, weight-adjusted-waist index (WWI), obesity, Alzheimer’s disease (AD), body mass index (BMI)

## Abstract

**Background:**

Obesity exerts a significant detrimental impact on cognitive function. The weight-adjusted waist index (WWI) serves as a more precise indicator of visceral obesity that is independent of weight, in contrast to body mass index (BMI). Still, little research has been conducted on the interrelation between WWI and cognitive abilities. This investigation explored the link between WWI and older Americans’ cognitive function.

**Methods:**

Utilizing data from the 2011–2014 National Health and Nutrition Examination Cross-Sectional Survey, multiple linear regression analysis was used to assess the influence of WWI on cognitive abilities in those over 60. Three cognitive assessments were administered: the Animal Fluency Test (AFT), the Digit Symbol Substitution Test (DSST), and the Word Learning and Recall Module from the Coalition to Establish a Registry for Alzheimer’s Disease (CERAD). We implemented threshold effects analysis and smoothed curve fitting to elucidate the nonlinear relationship. Additionally, we analyzed subgroups to check for variability.

**Results:**

There were 2,762 participants, including1,504 (54.45%) females and 1,258 (45.55%) males. 53.77% of them had completed high school or above, and their average age was 69.05 ± 6.62. After controlling for confounding variables, the improved model predicted a negative connection between WWI and CERAD-Total, AFT, and DSST scores (all *p* < 0.05). Furthermore, we performed a smoothenable curve fitting between WWI and cognitive function scores, resulting in a nonlinear connection and a threshold saturation effect. We also executed subgroup analyses and interaction tests based on gender, race, educational background, marital status, diabetes, body mass index, alcohol consumption, hypertension, smoking habits, stroke, depression, and sleep quality to assess whether the relationship between WWI and cognitive function was affected by heterogeneity across different population segments. The subgroup analysis found no significant differences in cognitive performance associated with WWI across the various subgroups.

**Conclusion:**

Higher WWI levels are associated with impaired cognitive function in Americans aged 60 and older.

## 1 Introduction

Cognitive decline is increasingly prevalent among the older adult demographic. Neurofibrillary tangles and amyloid plaques are recognized as the primary pathological hallmarks of Alzheimer’s disease (AD), a neurodegenerative disorder marked by increasing cognitive impairment, behavioral problems, and psychiatric problems (De-Paula et al., 2012). Currently, approximately 50 million individuals globally are affected by dementia, with projections indicating an increase to 80 million by 2030 (Cummings et al., 2016; Alzheimer’s Association., 2023). Some researchers predict that by 2050, the number of dementia patients will triple (Scheltens et al., 2021). Furthermore, the annual financial burden of caregiving for individuals with dementia is projected to reach $1 trillion worldwide (Breijyeh and Karaman, 2020). The disease has a growing socio-economic cost that increases in proportion to its prevalence, having an impact on families, society, and people to differing degrees and causing loss (Alzheimer’s Association., 2021). However, the condition cannot be prevented or its progression stopped; present treatments only alleviate its symptoms (Livingston et al., 2020; Yiannopoulou and Papageorgiou, 2020).

Obesity, a modifiable lifestyle determinant, has recently been associated with an elevated risk of cognitive decline and Alzheimer’s disease, particularly among middle-aged individuals with obesity. Early-stage lifestyle interventions, including weight reduction, enhanced nutritional practices, and increased physical activity, may mitigate disease risk and support the preservation of long-term cognitive function. Currently, obesity represents one of the most pressing global health challenges, with its prevalence escalating annually. The aetiology of obesity is multifactorial, involving a combination of genetic predispositions and behavioral as well as environmental influences (Heymsfield and Wadden, 2017). By 2030, projections indicate that over 50% of the global population will be overweight (Stecker and Sparks, 2006; Ward et al., 2019). We must thus address the obesity problem. Obesity and cognitive deterioration have been linked (Flores-Cordero et al., 2022). Numerous studies have demonstrated a relationship between body mass index (BMI) and cognitive deterioration (Nordestgaard et al., 2022; Costache et al., 2023). One investigation revealed a significant U-shaped association between BMI and dementia, indicating that both obesity and underweight status are linked to an increased likelihood of developing dementia (Beydoun et al., 2008). Traditionally, obesity has been assessed using body mass index (BMI). This metric focuses on the weight-to-height ratio but does not distinguish between muscle and fat composition or account for centripetal obesity, in which fat accumulates mainly in the stomach area (Hainer and Aldhoon-Hainerová, 2013; Javed et al., 2015). In contrast, the weight-adjusted waist index (WWI) incorporates waist circumference and body weight, effectively capturing changes in waist circumference attributable to variations in muscle or fat. Research has indicated that visceral fat is a more accurate indicator of the body’s metabolic status than BMI (Thomas et al., 2012). As a result, the weight-adjusted waist circumference index (WWI), a novel metric for assessing obesity, was proposed (Park et al., 2018).

WWI provides a more accurate representation of central obesity independent of body weight and better captures the extent of visceral obesity. It does this by normalizing body weight to waist circumference (WC), addressing the shortcomings of BMI, and incorporating the benefits of WC (Kim et al., 2021; Qin et al., 2022). Also, WWI has been linked to several health issues, including diabetes, high blood pressure, atherosclerosis, and coronary artery disease, according to a recent study (Li et al., 2020; Liu et al., 2021). Consequently, WWI provides a more nuanced analysis of body composition and serves as a more accurate metric for obesity, particularly about abdominal fat distribution. Nevertheless, there is a paucity of research exploring the association between WWI and cognitive function. To address this gap, we conducted cross-sectional studies utilizing data from the National Health and Nutrition Examination Survey (NHANES) spanning 2011 to 2014.

## 2 Methods

### 2.1 NHANES

This cross-sectional data was obtained through the National Health Statistics (NCHS), a division of the Centers for Disease Control and Prevention (CDC), which oversaw the design and implementation of the national survey known as NHANES (Johnson et al., 2014). The research adhered to the ethical principles established in the Helsinki Declaration of 1975. Each participant provided informed consent, and the project received approval from the NCHS Research Ethics Review Board. A variety of methodologies were employed, including laboratory analyses, in-person health assessments, surveys, and interviews, to evaluate the overall health, nutritional status, familial health history, and socioeconomic data of U.S. citizens. The participant cohort was representative, ensuring the reliability of the data across multiple health-related domains (Curtin et al., 2013). All information on the NHANES research design and data may be found at www.cdc.gov/nchs/nhanes. The observational research followed the STROBE principles for reporting cross-sectional studies (von Elm et al., 2007).

### 2.2 Study population

**Figure 1:**
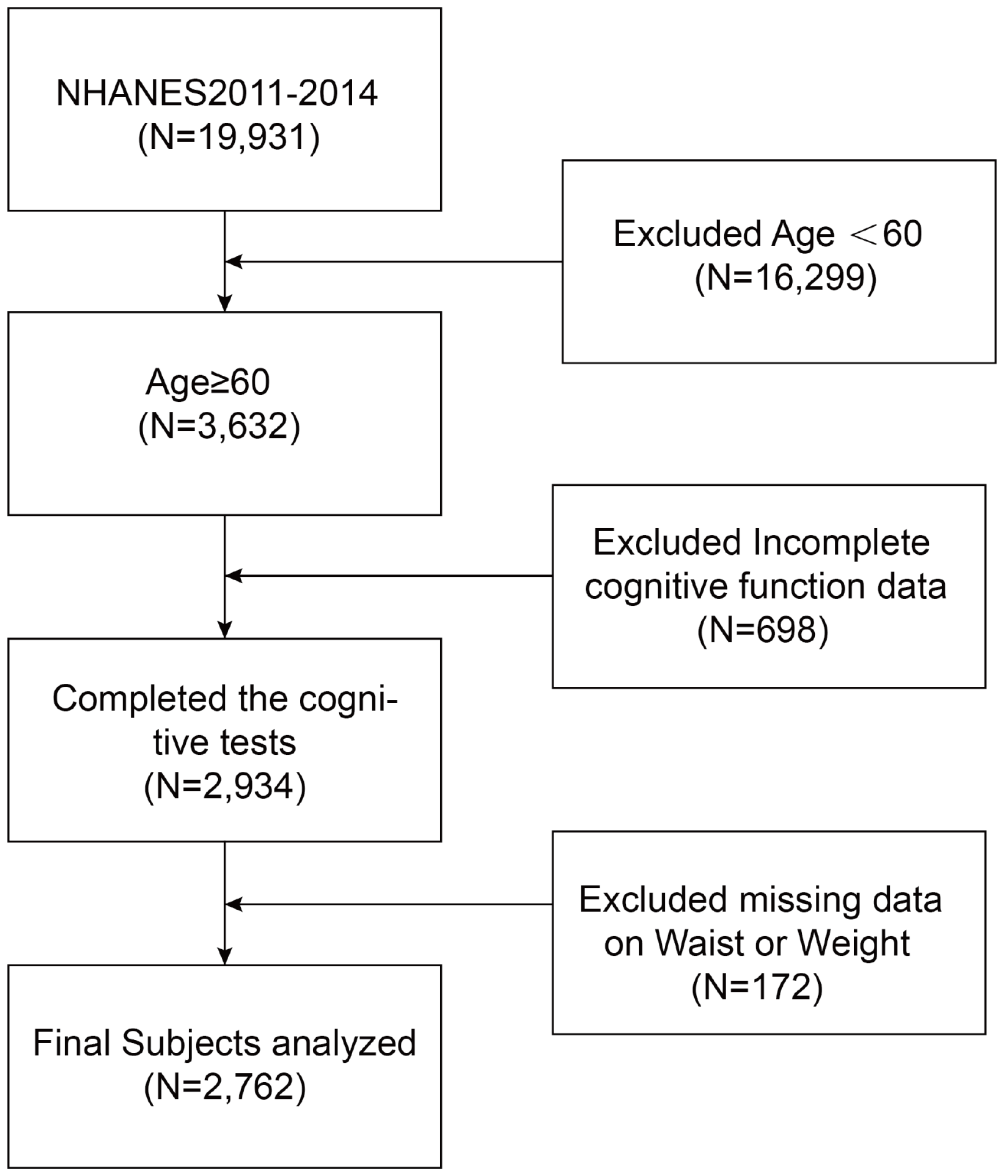
Flow chart of participants selection. NHANES, National Health and Nutrition Examination Survey.

Only 2011–2014 were used to produce cognitive function ratings in the NHANES research. As a result, we conducted our studies using this period. Given that the NHANES database primarily assesses cognitive performance in individuals aged 60 and older, our research specifically targeted this demographic. Our analyses included participants whose complete assessments of cognitive function and WWI data were available. In the NHANES database from 2011 to 2014, there were 19,931 participants, including 9,859 males (49.47%) and 10,072 females (50.53%).In the end, our analysis revealed 2,762 suitable adults in subsequent analyses after removing participants under 60 years old (*n* = 16,299), lacking any data about cognitive function (*n* = 698), and WWI (*n* = 172) (Figure 1). These 2,762 participants showed no statistical differences in gender, race, and education level compared to the 19,931participants above.

### 2.3 Weight-adjusted-waist index (WWI)

WWI is a distinct metric that outperforms BMI in assessing central obesity (Kim et al., 2021). It calculates using two variables: waist circumference and weight. Physical body weight and waist circumference measurements were taken in a mobile examination center by technicians who had received standard training. The WWI is calculated as the waist circumference (in centimeters) divided by square root of body weight (in kilograms) (Park et al., 2018). The resulting value is rounded to five decimal places. WWI was incorporated into this study as an exposure variable meriting further exploration. Given that WWI is a continuous variable, we categorized it into quartiles for subsequent analysis. The defined ranges for the WWI quartile groups are 9.02–11.00, 11.00–11.48, 11.48–11.94, and 11.94–14.79.

### 2.4 Assessment of cognitive functions

In the NHANES study, cognitive function was only measured during the 2011–2012 and 2013–2014 periods. Three cognitive assessments were used in the study: the Animal Fluency Test (AFT), the Digit Symbol Substitution Test (DSST), and the Word Learning and Recall Module from the Coalition to Establish a Registry for Alzheimer’s Disease (CERAD). Regarding the CERAD assessment, each component is designed to evaluate the participant’s capacity for rapid word acquisition and subsequent recall (Morris et al., 1989). The CERAD protocol consists of three word learning tasks followed by a single delayed recall assessment. NHANES personnel were instructed to audibly present 10 words to each participant. Following the presentation, participants endeavored to memorize and reproduce as many words as possible. The 10 words were administered in three different sequences. Each trial was scored from 0 to 10 points, and the cumulative scores from the three trials were aggregated to yield the immediate word learning total (CERAD-Total score ranging from 0 to 30). Furthermore, two additional cognitive tasks (AFT and DSST) were completed before administering the Delayed Recall Test (CERAD-delayed) (Fillenbaum et al., 2008). The Delayed Recall Test score is determined by the number of words recalled (CERAD-Delayed score 0–10). The Animal Fluency Test (AFT), which examines executive functions, focuses on categorical verbal fluency (Long et al., 2015). The sole requirement of the exam is that the test taker consciously speaks out the animal’s name; no prior educational background is necessary (Prince et al., 2003). The participant has 1 min to list as many animals as possible; they will receive one point for each species they can name (Wei et al., 2022). The Wechsler Adult Intelligence Scale (WAIS-III), which assesses memory, sustained focus, and response time, includes the Digit Symbol Substitution Test (DSST). Examinees have 1 min to accurately match each of the 133 boxes next to the numbers with the corresponding symbol. The score is the number of accurate matches (Jaeger, 2018).

### 2.5 Covariates

Possible confounding variables that could affect the relationship between WWI and cognitive performance were considered in the research. These variables encompassed sex, age, ethnicity, marital status, educational background, body mass index (BMI, kg/m2), PIR, creatinine concentration in serum (mg/dl), total cholesterol level in serum (mg/dl), high blood pressure, diabetes, smoking, alcohol intake, stroke, depression, and sleep disorder. Individuals were classified as smokers if they had smoked 100 or more cigarettes in their lifetime, and as drinkers if they had consumed 12 or more alcoholic beverages per year. The racial categories utilized comprised non-Hispanic whites, non-Hispanic blacks, Mexican Americans, other Hispanics, and additional racial groups, including multiracial individuals. Marital status was categorized based on whether individuals lived alone or cohabited. The four categories of educational achievement were beyond high school, high school diploma, ninth to eleventh grade, and lower than ninth grade. For further details on the subjects covered, check to www.cdc.gov/nchs/nhanes/.

### 2.6 Statistical analyses

All statistical analyses in this study obeyed CDC recommendations, and NHANES sample weights were used appropriately (Johnson et al., 2013). Categorical data were presented as percentages, while continuous variables were reported as means accompanied by standard deviations. Weighted Student’s *t*-tests (for continuous data) or weighted chi-square tests (for categorical data) assessed differences between groups divided into WWI quartiles. Meanwhile, we evaluated the following three models individually. Model 1 did not account for variables. Model 2 was only calibrated considering gender, age, and race. In contrast, Model 3 considered additional factors such as marital status, education, PIR, smoking habits, alcohol consumption, BMI, serum total cholesterol levels, serum creatinine levels, hypertension, diabetes, stroke, depression, and sleep disorder. Multiple linear regression analyses were utilized to explore the associations between WWI and cognitive outcomes, specifically CERAD-Total, CERAD-Delayed, AFT, and DSST across the three models. Participants’ BMI was categorized into quartiles. The relationships between WWI and cognitive abilities were further examined within subgroups defined by sex, ethnicity, educational attainment, marital status, BMI quartile classification, presence of diabetes or hypertension, smoking, alcohol intake, stroke, depression, and sleep disorder. To assess the heterogeneity of these relationships among subgroups, we incorporated additional interaction terms. Considering the interactions and connections between subgroups, we used R version 4.1.3 to conduct False Discovery Rate (FDR) Benjamini-Hochberg correction on the *p*-values for interactions. As nonlinear correlations were identified, a threshold analysis of effects was conducted with a log-likelihood ratio test to determine whether a threshold existed. To assure the study’s accuracy, all variables associated with the exposure variable WWI and the outcome variable cognitive function scores: weight, waist circumference, CERAD score, DSST score, and AFT score were removed if they were absent from the original data. Missing values for the other covariates were calculated using the median for continuous variables and the mode for categorical variables based on existing instances. Further, we used the VIF (Variance Inflation Factor) calculation to clarify potential multicollinearity issues among covariates. Conventionally, a variance inflation value (VIF) of less than 5 is acceptable. We calculated that all the relevant covariates mentioned in this study have VIF values less than 5, excluding potential multicollinearity issues among covariates. All statistical evaluations were conducted utilizing R version 4.1.3^1^ (R Foundation) and the Empower software^2^ (X&Y Solutions, Inc., Boston, MA).

## 3 Results

### 3.1 Baseline characteristics

The study comprised 2,762 participants, including 1,504 (54.45%)females and 1,258 (45.55%) males. Among these individuals, non-Hispanic white people make up 79.29%. 65.39% are cohabitation, and 53.77% have more education than a high school diploma. The average age of the participants was 69.05, with a standard deviation of 6.62. The individuals’ average cognitive function scores on the CERAD-Total, CERAD-Delayed, AFT, and DDST were 19.84 ± 4.43, 6.30 ± 2.26, 18.26 ± 5.67, 52.63 ± 16.52. Furthermore, we quartile WWI and the number of participants in each of its four groups is 691, 690, 690, and 691. As the quartiles of WWI increased, there was a decline in the scores of CERAD-Total, CERAD-delayed, AFT, and DDST from the four groups (*p* < 0.05). The four WWI quartile divisions demonstrated significant differences (all *p* < 0.05) in terms of age, sex, race, educational attainment, marital status, PIR, BMI, diabetes, alcohol consumption, hypertension, smoking, sleep disorder, stroke, cholesterol, CERAD-Total scores, CERAD-Delayed scores, AFT scores, and DDST scores (Table 1).

**Table 1:** Basic characteristics of participants by weight-adjusted-waist index quartiles among U.S. older adults.

Characteristics	WWI (quartile)					
	Overall *N* = 2,762	Q1 (9.02–11.00)	Q2 (11.00–11.48)	Q3 (11.48–11.94)	Q4 (11.94–14.79)	*P*-value
		*N* = 691	*N* = 690	*N* = 690	*N* = 691	
Age (years)	69.05 ± 6.62	67.21 ± 6.09	68.92 ± 6.65	69.72 ± 6.59	70.50 ± 6.68	<0.0001
Gender (%)						<0.0001
Male	1,258 (45.55%)	351 (50.84%)	343 (49.73%)	336 (48.64%)	228 (33.03%)	
Female	1,504 (54.45%)	340 (49.16%)	347 (50.27%)	354 (51.36%)	463 (66.97%)	
Race,(%)						0.0007
Mexican American	97 (3.51%)	13 (1.90%)	22 (3.17%)	31 (4.39%)	31 (4.53%)	
Other Hispanic	106 (3.84%)	12 (1.64%)	32 (4.69%)	25 (3.65%)	37 (5.28%)	
Non-Hispanic White	2,190 (79.29%)	554 (80.22%)	546 (79.13%)	550 (79.68%)	540 (78.16%)	
Non-Hispanic Black	227 (8.22%)	75 (10.89%)	58 (8.47%)	49 (7.07%)	45 (6.53%)	
Other race-including multi-racial	142 (5.15%)	37 (5.35%)	32 (4.54%)	35 (5.22%)	38 (5.50%)	
Education level (%)						<0.0001
Less than 9th grade	993 (35.95%)	311 (45.03%)	245 (35.56%)	227 (32.91%)	210 (30.37%)	
9–11th grade	284 (10.28%)	55 (7.87%)	63 (9.09%)	68 (9.79%)	98 (14.22%)	
High school	608 (22.02%)	124 (17.97%)	149 (21.63%)	160 (23.22%)	175 (25.33%)	
﹥High school	877 (31.75%)	201 (29.13%)	233 (33.73%)	235 (34.08%)	208 (30.08%)	
Marital status						<0.0001
Cohabitation	1806 (65.39%)	493 (71.38%)	500 (72.43%)	436 (63.16%)	377 (54.58%)	
Solitude	956 (34.61%)	198 (28.62%)	190 (27.57%)	254 (36.84%)	314 (45.42%)	
Diabetes (%)						<0.0001
Yes	534 (19.33%)	63 (9.09%)	93 (13.43%)	139 (20.20%)	239 (34.63%)	
No	2,228 (80.67%)	628 (90.91%)	597 (86.57%)	551 (79.80%)	452 (65.37%)	
Alcohol intake ≧ 12 drinks/year (%)						<0.0001
Yes	2021 (73.17%)	540 (78.12%)	515 (74.65%)	542 (78.50%)	424 (61.42%)	
No	741 (26.83%)	151 (21.88%)	175 (25.35%)	148 (21.50%)	267 (38.58%)	
HBP (%)						<0.0001
Yes	1,611 (58.33%)	315 (45.60%)	367 (53.12%)	443 (64.21%)	486 (70.28%)	
No	1,151 (41.67%)	376 (54.40%)	323 (46.88%)	247 (35.79%)	205 (29.72%)	
Smoking (%)						0.0231
Yes	1,400 (50.69%)	319 (46.17%)	374 (54.20%)	357 (51.68%)	350 (50.65%)	
No	1,362 (49.31%)	372 (53.83%)	316 (45.80%)	333 (48.32%)	341 (49.35%)	
Depression (%)						0.1439
No	2,102 (76.10%)	538 (77.95%)	551 (79.79%)	518 (75.09%)	495 (71.59%)	
Mild	542 (19.63%)	129 (18.60%)	115 (16.70%)	142 (20.64%)	156 (22.66%)	
Moderate	90 (3.26%)	23 (3.29%)	17 (2.49%)	21 (3.03%)	29 (4.11%)	
Severe	28 (1.01%)	1 (0.16%)	7 (1.01%)	9 (1.23%)	11 (1.64%)	
Sleep disorder (%)						0.0002
Yes	876 (31.72%)	190 (27.54%)	198 (28.74%)	228 (33.10%)	260 (37.62%)	
No	1886 (68.28%)	501 (72.46%)	492 (71.26%)	462 (66.90%)	431 (62.38%)	
Stroke (%)						0.0012
Yes	162 (5.87%)	35 (5.00%)	29 (4.21%)	37 (5.29%)	61 (8.88%)	
No	2,600 (94.13%)	656 (95.00%)	661 (95.79%)	653 (94.71%)	630 (91.12%)	
Family PIR	3.14 ± 1.58	3.54 ± 1.53	3.32 ± 1.53	3.10 ± 1.54	2.53 ± 1.53	<0.0001
BMI	28.97 ± 6.15	26.02 ± 4.97	28.28 ± 5.21	29.88 ± 6.26	31.95 ± 6.48	<0.0001
Cholesterol (mg/dL)	192.56 ± 43.22	197.97 ± 40.24	189.81 ± 43.84	192.86 ± 45.97	189.11 ± 42.11	0.0006
Creatinine (mg/dL)	0.98 ± 0.46	0.98 ± 0.59	0.98 ± 0.40	0.98 ± 0.39	1.00 ± 0.41	0.8317
CERAD-total	19.84 ± 4.43	20.68 ± 4.37	19.78 ± 4.59	19.63 ± 4.22	19.19 ± 4.42	<0.0001
CERAD-delayed	6.30 ± 2.26	6.58 ± 2.11	6.30 ± 2.31	6.19 ± 2.28	6.11 ± 2.31	0.0005
AFT	18.26 ± 5.67	19.16 ± 5.82	18.46 ± 5.52	18.14 ± 5.44	17.19 ± 5.71	<0.0001
DDST	52.63 ± 16.52	56.71 ± 16.12	53.26 ± 16.21	52.81 ± 15.91	47.35 ± 16.47	<0.0001

Mean ± SD for continuous variables: the *p*-value was calculated by the weighted linear regression model; (%) for categorical variables: the *p*-value was calculated by the weighted chi-square test. PIR, the ratio of income to poverty; BMI, body mass index; CERAD, Consortium to Establish a Registry for Alzheimer’s Disease; AFT, Animal Fluency test; DSST, Digit Symbol Substitution test; Q, quartiles; HBP, high blood pressure.

### 3.2 Correlation between WWI and cognition test results

Table 2 compellingly illustrates the relationship between WWI and cognitive performance. The fully modified model revealed that the three cognitive function scores (CERAD-Total, AFT, and DDST) dropped by 0.48, 0.50, and 1.71 units for each unit of increase in the WWI score. After that, the WWI data was divided into quartiles to conduct additional sensitivity testing. Furthermore, in Model 1 (the original model) and Model 2 (the model with moderate adjustments), the *β*-values for the CERAD-Total scores of participants in the highest quartile (Q4) compared to those in the lowest quartile (Q1) were-1.49 and-0.98, respectively. The 95% confidence intervals were-1.95 to-1.02 and-1.42 to-0.54, and their *p*-values for the trend were less than 0.0001. This implies that the linked CERAD-Total scores fell as the WWI quartile groupings rose in Models 1 and 2, with a significant trend. In Model 1, compared to the lowest WWI quartile (Q1), the *β*-values for the CERAD-Delayed scores of individuals in the highest WWI quartile (Q4) was –0.47. The 95% confidence interval was –0.71 to –0.23, and the *p*-values for the trend were less than 0.0001. This suggests that CERAD-Delayed scores had a declining and statistically significant negative trend as the WWI quartile groups kept rising in Model 1. In both Model 1 and Model 2, people in the highest quartile of WWI (Q4) demonstrated lower AFT scores than those in the lowest quartile of WWI (Q1). Their β-values were –1.97 and –1.03, respectively, with 95% CIs of –2.56 to –1.38, −1.59 to –0.46, and the *p*-values for the trend were less than 0.0001 and 0.0004. This suggests that their associated AFT scores in Models 1 and 2 fall steadily and significantly as the WWI quartile groups grow. Regarding DDST scores, Model 1 and Model 2 showed significant trend testing. The *β*-values for DSST scores of participants in the highest WWI quartile (Q4) were –9.63 and –6.51, respectively, with 95% confidence intervals of –11.07 to –7.66 and –7.98 to –5.04. Their *p*-value for the trend was less than 0.0001. Overall, the four cognitive function scores reflect a consistent decrease as WWI increases, suggesting that lower cognitive function scores may be associated with higher WWI.

**Table 2:** Association between WWI and cognitive function scores.

Model			Model1 β (95%CI)	*P*-value	Model2 β (95%CI)	*P*-value	Model3 *β* (95%CI)	*P*-value
CERAD--total	WWI		−0.91 (−1.15, −0.68)	<0.0001	−0.63 (−0.85, −0.40)	<0.0001	−0.48 (−0.81, −0.15)	0.0050
	WWI (quartile)	Q1	Ref		Ref		Ref	
		Q2	−0.90 (−1.36, −0.44)	0.0001	−0.49 (−0.92, −0.06)	0.0263	−0.63 (−1.21, −0.04)	0.0357
		Q3	−1.05 (−1.51, −0.59)	<0.0001	−0.50 (−0.93, −0.07)	0.0228	−0.30 (−0.90, 0.31)	0.3346
		Q4	−1.49 (−1.95, −1.02)	<0.0001	−0.98 (−1.42, −0.54)	<0.0001	−0.71 (−1.35, −0.08)	0.0277
		*P* for trend	<0.0001		<0.0001		0.0639	
CERAD--delayed	WWI		−0.33 (−0.45, −0.21)	<0.0001	−0.19 (−0.30, −0.07)	0.0017	−0.14 (−0.32, 0.03)	0.1111
	WWI (quartile)	Q1	Ref		Ref		Ref	
		Q2	−0.28 (−0.52, −0.05)	0.0189	−0.08 (−0.30, 0.14)	0.4834	−0.13 (−0.44, 0.17)	0.3866
		Q3	−0.40 (−0.63, −0.16)	0.0010	−0.12 (−0.34, 0.10)	0.2758	0.02 (−0.29, 0.34)	0.8870
		Q4	−0.47 (−0.71, −0.23)	0.0001	−0.22 (−0.44, 0.01)	0.0639	−0.14 (−0.47, 0.19)	0.4178
		*P* for trend	<0.0001		0.0595		0.5709	
AFT	WWI		−1.17 (−1.47, −0.87)	<0.0001	−0.65 (−0.94, −0.36)	<0.0001	−0.50 (−0.92, −0.09)	0.0175
	WWI (quartile)	Q1	Ref		Ref		Ref	
		Q2	−0.70 (−1.29, −0.11)	0.0199	−0.22 (−0.77, 0.33)	0.4335	−0.01 (−0.74, 0.71)	0.9689
		Q3	−1.02 (−1.61, −0.43)	0.0006	−0.37 (−0.92, 0.18)	0.1849	0.41 (−0.34, 1.16)	0.2866
		Q4	−1.97 (−2.56, −1.38)	<0.0001	−1.03 (−1.59, −0.46)	0.0004	−0.23 (−1.02, 0.56)	0.5655
		*P* for trend	<0.0001		0.0004		0.7153	
DDST	WWI		−5.14 (−6.00, −4.28)	<0.0001	−3.56 (−4.31, −2.81)	<0.0001	−1.71 (−2.73, −0.69)	0.0010
	WWI (quartile)	Q1	Ref		Ref		Ref	
		Q2	−3.45 (−5.15, −1.76)	<0.0001	−1.39 (−2.82, 0.03)	0.0556	−0.68 (−2.46, 1.09)	0.4509
		Q3	−3.91 (−5.59, −2.23)	<0.0001	−1.30 (−2.72, 0.13)	0.0740	1.93 (0.09, 3.77)	0.0399
		Q4	−9.36 (−11.07,-7.66)	<0.0001	−6.51 (−7.98, −5.04)	<0.0001	−1.85 (−3.78, 0.08)	0.0607
		*P* for trend	<0.0001		<0.0001		0.1957	

Q, quartiles; CI, confidence interval; Ref, reference. Model 1 was unadjusted. Model 2 was adjusted for age, gender, race. Model 3 was adjusted for age, gender, race, marital status, education, PRI, smoking status, drinking status, BMI, serum total cholesterol level, serum creatinine level, hypertension, diabetes, stroke, depression, sleep disorder.

### 3.3 Relationship between WWI and cognitive function scores

**Figure 2:**
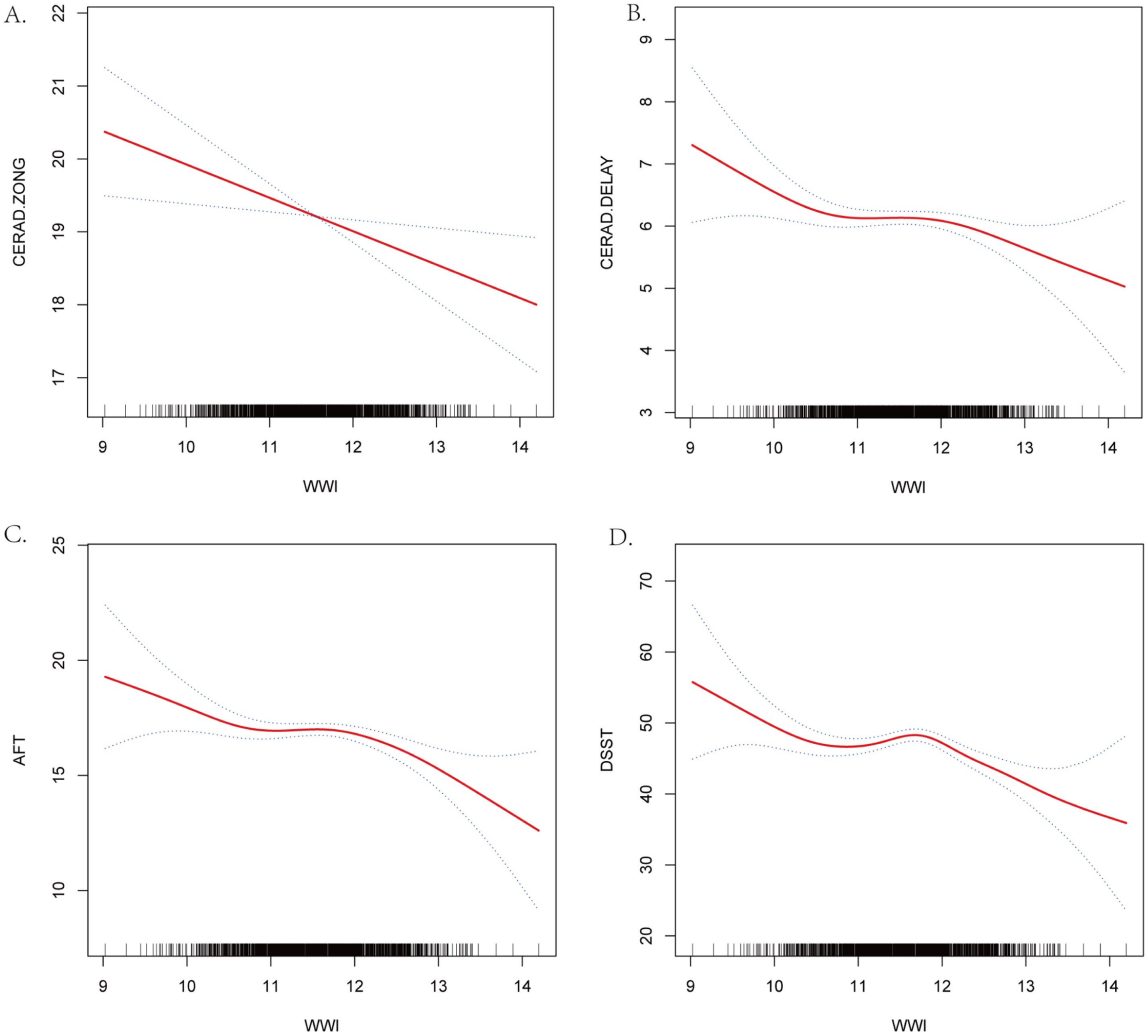
Smooth Curve Fitting Detected the nonlinear relationship between WWI and cognitive function scores. **(A)** CERAD-Total scores. **(B)** CERAD-Delayed scores. **(C)** AFT. **(D)** DSST. Adjusted for age, gender, race, marital status, education, PRI, smoking status. Drinking status, BMI, serum total cholesterol level, serum creatinine level, hyper-tension, diabetes, stroke, depression and sleep disorder.

Smoothed curve fitting was used to represent the association between WWI and cognitive function scores graphically. Figure 2 demonstrates that the four cognition scores, CERAD-Total scores, CERAD-Delayed scores, AFT scores, and DDST scores have a nonlinear correlation to WWI. Additional threshold effect analysis revealed breakpoints in the association between the two cognitive function scores (AFT and DDST) and WWI. The predicted breakpoint (K) in the nonlinear correlation between AFT scores and WWI is 12.67. There was no significant statistical association between WWI and AFT scores on the left side of the breakpoint, with the *β* value of –0.44, the 95% confidence interval of –0.89 to 0.02, and the *p*-value of 0.0587. A negative connection was observed between WWI and AFT scores on the right side of the breakpoint (*β* = −3.27, 95% CI –6.08 to –0.47; *p*-value = 0.0224), with a log-likelihood ratio test of 0.021. The nonlinear correlation between WWI and DDST scores demonstrates a breakpoint (K) of 11.81. A negative connection was observed between WWI and DSST scores on both sides of the breakpoint (The left side: *β* = −5.79, 95% CI –8.07 to –3.51, *p*-value < 0.0001; The right side: *β* = −6.36, 95% CI –9.50 to −3.22, *p*-value<0.0001), with a log-likelihood ratio test less than 0.001 (Table 3). The smoothed curve fitting of the WWI to the four categories of cognitive function scores revealed that the DSST was the most stable regarding WWI, with a consistent decreasing trend of acquisition at both the right and left ends of the inflexion point of 11.81. The DDST may represent the most sensitive indicator of cognitive performance within the NHANES dataset.

**Table 3:** Threshold effect analysis for correlations between the WWI and cognitive function scores.

CERAD-total	Inflection point(K)	10.52
	β(95%CI) (﹤K), *P-*value	−1.88 (−3.81, 0.06) 0.0573
	β(95%CI) (﹥K), *P-*value	−0.34 (−0.72, 0.04) 0.0787
	LLR	0.140
CERAD-delayed	Inflection point(K)	10.48
	β(95%CI) (﹤K), *P-*value	−1.20 (−2.23, −0.16) 0.0239
	β(95%CI) (﹥K), *P-*value	−0.14 (−0.33, 0.05) 0.1627
	LLR	0.056
AFT	Inflection point (K)	12.67
	β(95%CI) (﹤K), *p-*value	−0.44 (−0.89, 0.02) 0.0587
	β(95%CI) (﹥K), *p-*value	−3.27 (−6.08, −0.47) 0.0224
	LLR	0.021
DSST	Inflection point (K)	11.81
	β(95%CI) (﹤K), *p-*value	−5.79 (−8.07, −3.51) <0.0001
	β(95%CI) (﹥K), *p-*value	−6.36 (−9.50, −3.22) <0.0001
	LLR	<0.001

LLR, logarithmic likelihood ratio test *p*-value. Adjusted for age, gender, race, marital status, education, PRI, smoking, alcohol intake, BMI, total cholesterol level in serum, serum creatinine level in serum, hypertension, diabetes, stroke, depression and sleep disorder.

### 3.4 Subgroup analyses

To identify potentially heterogeneous populations and determine whether the associations between WWI and the four cognitive function scores—CERAD-Total, CERAD-Delayed, AFT, and DDST—were consistent across the population, we conducted analyses stratified by the following subgroups: gender, race, educational background, marital status, diabetes, BMI, alcohol intake, hypertension, smoking habits, stroke, depression, and sleep disorder. In the subgroup analysis, the FDR-adjusted *p*-values for interaction show that there was no interaction between WWI and cognitive function across subgroups (Table 4). This suggests that the negative relationship between WWI and cognitive function remains unaffected by the interactions of these subgroups. The negative association is both independent and robust.

**Table 4:** Subgroup analysis of the association between WWI and cognitive function scores.

Subgroup	CERAD--total β (95%CI) *p-value*	*P* for interac tion	FDR-adjusted *p*-values	CERAD--delayed β (95%CI) *p-value*	*P* for interaction	FDR-adjusted *p*-values	AFT β (95%CI) *p-value*	*P* for interaction	FDR-adjusted *p*-values	DDST β (95%CI)	*P* for interaction	FDR-adjusted *P*-values
Gender		0.7169	0.9791		0.7979	0.8704		0.0775	0.1862		0.3069	0.5261
Male	−0.55 (−1.17, 0.08) 0.0849			−0.08 (−0.40, 0.24) 0.6325			0.05 (−0.72, 0.82) 0.8897			−2.53 (−4.41, −0.64) 0.0088		
Female	−0.41 (−0.82, −0.01) 0.0463			−0.13 (−0.34, 0.08) 0.2306			−0.76 (−1.26, −0.26) 0.0029			−1.37 (−2.59, −0.14) 0.0290		
Race		0.9131	0.9791		0.9256	0.9256		0.9148	0.9148		0.9531	0.9531
Mexican American	0.46 (−1.64, 2.55) 0.6697			0.04 (−1.05, 1.13) 0.9400			−0.78 (−3.37, 1.81) 0.5556			−1.64 (−7.92, 4.65) 0.6096		
Other Hispanic	−0.37 (−2.41, 1.67) 0.7232			0.13 (−0.94, 1.20) 0.8101			0.27 (−2.26, 2.81) 0.8327			0.15 (−5.99, 6.29) 0.9616		
Non-Hispanic White	−0.51 (−0.88, −0.14) 0.0077			−0.12 (−0.31, 0.08) 0.2323			−0.47 (−0.94, −0.01) 0.0463			−1.73 (−2.85, −0.61) 0.0026		
Non-Hispanic Black	−0.51 (−1.77, 0.76) 0.4322			−0.37 (−1.03, 0.29) 0.2772			−0.98 (−2.55, 0.58) 0.2191			−0.88 (−4.68, 2.92) 0.6500		
Other Race - including multi-racial	−0.17 (−2.02, 1.68) 0.8604			−0.09 (−1.06, 0.88) 0.8544			−0.09 (−2.38, 2.21) 0.9405			−2.68 (−8.23, 2.88) 0.3451		
Education level (%)		0.4931	0.9791		0.3874	0.5811		0.1489	0.2978		0.0301	0.1806
Less than 9th grade	−0.64 (−1.25, −0.03) 0.0409			−0.04 (−0.36, 0.28) 0.7897			0.22 (−0.53, 0.97) 0.5618			−1.01 (−2.83, 0.81) 0.2759		
9-11th grade	0.04 (−0.84, 0.92) 0.9253			0.10 (−0.36, 0.56) 0.6750			−0.23 (−1.30, 0.84) 0.6693			1.46 (−1.15, 4.07) 0.2724		
High school	−0.30 (−1.02, 0.42) 0.4085			−0.37 (−0.74, 0.01) 0.0549			−1.09 (−1.97, −0.22) 0.0146			−3.43 (−5.56, −1.29) 0.0017		
﹥High school	−0.68 (−1.29, −0.07) 0.0291			−0.18 (−0.50, 0.14) 0.2726			−0.35 (−1.09, 0.40) 0.3631			−1.82 (−3.64, −0.00) 0.0498		
Marital status		0.8542	0.9791		0.4696	0.6261		0.2108	0.3614		0.0146	0.1752
Cohabitation	−0.42 (−0.85, 0.01) 0.0570			−0.06 (−0.29, 0.16) 0.5888			−0.24 (−0.78, 0.29) 0.3780			−0.59 (−1.91, 0.72) 0.3777		
Solitude	−0.48 (−1.01, 0.05) 0.0746			−0.19 (−0.47, 0.08) 0.1747			−0.77 (−1.43, −0.12) 0.0212			−3.15 (−4.77, −1.53) 0.0001		
Diabetes		0.1173	0.4692		0.0551	0.3031		0.0776	0.1862		0.1598	0.4794
Yes	0.06 (−0.71, 0.83) 0.8818			0.21 (−0.19, 0.61) 0.3009			0.24 (−0.71, 1.18) 0.6270			−0.05 (−2.39, 2.30) 0.9689		
No	−0.62 (−0.99, −0.24) 0.0013			−0.22 (−0.41, −0.02) 0.0286			−0.70 (−1.16, −0.24) 0.0030			−1.89 (−3.03, −0.75) 0.0012		
Alcohol intake ≧ 12drinks/year		0.7121	0.9791		0.6548	0.7858		0.0095	0.0528		0.2624	0.5261
Yes	−0.41 (−0.81, −0.01) 0.0433			−0.11 (−0.32, 0.10) 0.3175			−0.11 (−0.60, 0.39) 0.6752			−1.19 (−2.41, 0.04) 0.0578		
No	−0.54 (−1.12, 0.03) 0.0641			−0.19 (−0.49, 0.11) 0.2188			−1.22 (−1.93, −0.51) 0.0008			−2.38 (−4.13, −0.63) 0.0079		
HBP		0.9791	0.9791		0.1904	0.3808		0.0132	0.0528		0.5889	0.8834
Yes	−0.41 (−0.83, 0.02) 0.0598			−0.21 (−0.43, 0.02) 0.0701			−0.79 (−1.32, −0.26) 0.0034			−2.12 (−3.43, −0.81) 0.0015		
No	−0.42 (−0.96, 0.13) 0.1342			0.03 (−0.25, 0.32) 0.8264			0.28 (−0.40, 0.96) 0.4202			−1.55 (−3.23, 0.14) 0.0723		
Smoking		0.0218	0.2616		0.0956	0.3031		0.0096	0.0528		0.2804	0.5261
Yes	−0.05 (−0.53, 0.44) 0.8429			0.02 (−0.23, 0.28) 0.8611			0.12 (−0.48, 0.72) 0.6989			−0.99 (−2.48, 0.49) 0.1904		
No	−0.82 (−1.28, −0.36) 0.0005			−0.27 (−0.51, −0.03) 0.0279			−0.96 (−1.54, −0.39) 0.0010			−2.11 (−3.52, −0.70) 0.0035		
BMI (quartile)		0.7516	0.9791		0.1263	0.3031		0.7165	0.7816		0.8272	0.9531
Q1	−0.25 (−0.95, 0.46) 0.4954			0.02 (−0.35, 0.38) 0.9276			−0.76 (−1.63, 0.10) 0.0838			−2.48 (−4.62, −0.34) 0.0234		
Q2	−0.71 (−1.41, −0.01) 0.0470			−0.35 (−0.71, 0.01) 0.0584			−0.19 (−1.05, 0.66) 0.6578			−1.37 (−3.50, 0.75) 0.2060		
Q3	−0.28 (−1.01, 0.45) 0.4524			0.13 (−0.25, 0.51) 0.4948			−0.33 (−1.22, 0.56) 0.4677			−1.23 (−3.44, 0.99) 0.2776		
Q4	−0.54 (−1.20, 0.12) 0.1099			−0.34 (−0.68, −0.00) 0.0475			−0.72 (−1.52, 0.08) 0.0796			−1.99 (−3.99, 0.00) 0.0504		
Depression		0.1571	0.4713		0.0179	0.2148		0.3848	0.4618		0.9459	0.9531
No	−0.42 (−0.81, −0.03) 0.0329			−0.02 (−0.22, 0.18) 0.8151			−0.47 (−0.94, 0.00) 0.0524			−1.39 (−2.57, −0.21) 0.0207		
Mild	−1.09 (−1.85, −0.33) 0.0049			−0.66 (−1.06, −0.27) 0.0011			−0.69 (−1.62, 0.24) 0.1480			−1.91 (−4.23, 0.41) 0.1077		
Moderate	1.32 (−1.18, 3.83) 0.3008			0.67 (−0.63, 1.97) 0.3126			2.08 (−1.00, 5.16) 0.1853			−2.90 (−10.55, 4.74) 0.4569		
Severe	−6.29 (−23.93, 11.35) 0.4847			−1.53 (−10.70, 7.64) 0.7442			1.55 (−20.14, 23.24) 0.8885			−9.01 (−62.90, 44.88) 0.7432		
Sleep disorder		0.0896	0.4692		0.1155	0.3031		0.3119	0.4180		0.7915	0.9531
Yes	−0.09 (−0.65, 0.46) 0.7411			0.04 (−0.25, 0.33) 0.7852			−0.76 (−1.45, −0.07) 0.0319			−1.85 (−3.57, −0.14) 0.0341		
No	−0.69 (−1.10, −0.27) 0.0012			−0.24 (−0.46, −0.03) 0.0262			−0.32 (−0.83, 0.19) 0.2182			−1.57 (−2.84, −0.30) 0.0154		
Stroke		0.7132	0.9791		0.3264	0.5595		0.3135	0.4180		0.0535	0.2140
Yes	−0.76 (−2.09, 0.58) 0.2670			0.16 (−0.53, 0.86) 0.6458			−1.34 (−3.00, 0.32) 0.1134			−5.57 (−9.64, −1.49) 0.0075		
No	−0.50 (−0.85, −0.16) 0.0045			−0.19 (−0.37, −0.01) 0.0378			−0.47 (−0.90, −0.04) 0.0307			−1.49 (−2.54, −0.43) 0.0058		

Age, Gender, Race, marital status, education, PRI, smoking, alcohol intake, BMI, total cholesterol level in serum, serum creatinine level in serum, hypertension, diabetes, stroke, depression and sleep disorder were adjusted. FDR, false discovery rate.

## 4 Discussion

This research aimed to explore the relationship between the cognitive abilities of US citizens and their assessments of WWI. In this cross-sectional investigation, which included 2,762 participants, we observed an extent link between WWI and these four cognitive function scores. This suggests a pertinence between lower cognitive function scores and increased WWI. However, a non-linear accordance emerged between WWI and the four cognitive function scores. In summary, the WWI index may serve as a valuable metric for assessing cognitive capacity.

Previous research has indicated a potential association between obesity and cognitive functioning (Sturman et al., 2008; Smith et al., 2014; Gunathilake et al., 2016). Notably, our analysis of substantial population-based samples revealed that childhood obesity adversely affects cognitive abilities in adulthood (Zhou et al., 2020). The Eastern Finland Cardiovascular Risk Factors, Aging, and Dementia (CAIDE) project examined the relationship between body mass index (BMI) in midlife and late life and the subsequent onset of dementia. This extensive longitudinal study tracked 1,304 participants over a span of 26 years. Findings suggest that an elevated BMI during midlife significantly increases the risk of developing dementia (Tolppanen et al., 2014; Zhang et al., 2021; den Brok et al., 2022). A cohort study involving elderly African Americans in the Chicago area established a link between cognitive decline and elevated BMI, indicating that greater fluctuations in BMI correlate with accelerated cognitive impairment. Consequently, it is imperative to focus on the impact of stable BMI on cognitive function (Aiken-Morgan et al., 2020). In addition to prior investigations into the causal relationship between BMI and cognitive abilities, a U.S. cohort study also assessed waist circumference. The results demonstrated a significant association between increased waist circumference and a more rapid cognitive decline, whereas no substantial link was found between BMI and cognitive deterioration (West et al., 2017). Evidence suggests that individuals with moderate obesity may have a more favorable prognosis than those with a normal BMI concerning heart failure, acute coronary syndromes, and coronary artery disease (Elagizi et al., 2018; Park et al., 2018; Ding et al., 2022). Numerous studies have reported inconsistent relationships between cognitive function and BMI. One investigation identified a negative correlation between BMI and cognitive capacity, indicating that higher BMI is associated with poorer cognitive performance (Benito-León et al., 2013). Conversely, another study revealed a significant U-shaped relationship between BMI and dementia, with both obesity and underweight status linked to an elevated risk of dementia (Beydoun et al., 2008). This phenomenon is often referred to as the “obesity paradox” concerning BMI. Thus, investigations linking WWI to cognition were warranted, given these inconsistent BMI-cognition findings. The WWI, which essentially indicates the presence of visceral fat, was found to have a negative connection with cognitive abilities in older adults in another cohort investigation (Rodríguez-Fernández et al., 2017; Kim et al., 2022). In this investigation, we utilized BMI as a covariate to account for the possible confounding factor of weight and height on the link between visceral obesity and cognitive performance.

It is essential to explore the various pathways that connect visceral obesity to cognitive decline. Insulin resistance and metabolic syndrome can be caused by various factors, including obesity (Sędzikowska and Szablewski, 2021). Insulin drives changes in hippocampus synaptic plasticity, affecting catecholamine neurotransmitter synthesis and absorption, maintaining neuronal cell proliferation, and balancing the body’s glucose levels (Schrijvers et al., 2010; Gratuze et al., 2018). Insulin may easily permeate the blood–brain barrier. However, the body’s insulin resistance causes insulin signaling pathway disruptions, speeding up neurons’ degeneration and lowering cognitive performance (van der Heide et al., 2006; Nelson et al., 2008; Arnold et al., 2018). Fat tissue emits inflammatory substances like IL-1β, IL-6, MCP1, and C-reactive protein (Dermitzaki et al., 2014). These inflammatory mediators can cause a low-grade, chronic inflammatory state, impairing cognitive function and promoting dementia. Additionally, obesity often leads to leptin resistance, disrupting signalling and regulatory mechanisms and negatively affecting cognitive function (Poulos et al., 2010; Forny-Germano et al., 2018; Flores-Cordero et al., 2022; Nozari et al., 2023).

This research elucidates the relationship between WWI and the risk of cognitive decline in the elderly, offering fresh insights into the early detection and prevention of cognitive deterioration. The nonlinear relationship between WWI and cognitive function found in the study, especially the increased risk of cognitive decline at high WWI values, provides new possibilities for exploring the threshold point for the effect of obesity on cognitive function. These findings are instructive for public health policymakers, emphasizing the importance of reducing obesity and improving cognitive health to help develop effective public health strategies. The study’s particular focus on the elderly population suggests that future research could target this population to create specific nutritional and lifestyle interventions to slow cognitive decline. WWI is a novel metric for measuring obesity that allows for more exact measurements of muscle and fat mass. This conclusion recommends that future research investigate the implications of various body composition measures on cognitive performance and ways to prevent or halt cognitive decline by increasing these metrics. When combined with WWI’s superiority, DEXA (Dual Energy X-ray Absorptiometry) is a beneficial tool for assessing older persons’ body composition and cognitive function. DEXA makes it possible to measure and distribute body fat and lean tissue and accurately evaluate bone mineral density and other aspects of body composition. DEXA measures offer a more accurate evaluation of lean tissue (such as muscle mass) and fat distribution than other techniques since muscle mass is often lower in older persons. This is especially crucial when examining the connection between body composition and cognitive performance. The association between body fat and muscle mass, as well as their distribution and cognitive function in older persons, may be examined using data from DEXA.

The current research offers several benefits. First, WWI is relatively new and innovative compared to earlier studies and more accurately responds to visceral obesity. It is supported by better research and offers new insights into the correlation between fat and the assessment of cognition. Secondly, 2,417 nationally representative persons selected by normative sampling participated in our study. The large enough sample size enables additional subgroup analyses to explore the association between WWI and cognition in various demographic and subgroup contexts. This improves the statistical validity and stability of the study. However, this study has limitations. Initially, this analysis was conducted based on the NHANES public records. Due to its cross-sectional design, it is limited in examining the causal connection and can only evaluate the degree of the relationship between WWI and cognitive function. A large sample size and a prospective investigation will be needed to confirm this causal association. Secondly, some confounding variables such as diet quality and physical activity could still impact the study’s findings, even though we adjusted for most covariates that might affect the results. Additionally, the NHANES database originates from the United States, and all research participants are American citizens; therefore, additional validation of our findings is required for other nations.

## 5 Conclusion

Our investigation came to the following conclusion: the cognitive function scores decreased with increasing WWI. Meanwhile, their interaction was nonlinear. In addition, WWI may be used as a reference index for evaluating cognitive function in people over 60.

## Data Availability

The datasets presented in this study can be found in online repositories. The names of the repository/repositories and accession number(s) can be found at: https://wwwn.cdc.gov/nchs/nhanes.

## Footnotes

^1^ http://www.R-project.org

^2^ www.empowerstats.com
